# Comparison of Thermal Detector Arrays for Off-Axis THz Holography and Real-Time THz Imaging

**DOI:** 10.3390/s16020221

**Published:** 2016-02-06

**Authors:** Erwin Hack, Lorenzo Valzania, Gregory Gäumann, Mostafa Shalaby, Christoph P. Hauri, Peter Zolliker

**Affiliations:** 1 Reliability Science and Technology Laboratory, Empa, Überlandstrasse 129, CH-8600 Dübendorf, Switzerland; lorenzo.valzania@empa.ch (L.V.); peter.zolliker@empa.ch (P.Z.); 2 Institute of Applied Physics, University of Bern, Sidlerstrasse 5, CH-3012 Bern, Switzerland; gregory.gaeumann@iap.unibe.ch; 3 SwissFEL Laser Group, Paul Scherrer Institute, CH-5232 Villigen PSI, Switzerland; mostafa.shalaby@psi.ch (M.S.); christoph.hauri@psi.ch (C.P.H.)

**Keywords:** terahertz, digital holography, array detector, micro-bolometer, pyroelectric detector, real time imaging

## Abstract

In terahertz (THz) materials science, imaging by scanning prevails when low power THz sources are used. However, the application of array detectors operating with high power THz sources is increasingly reported. We compare the imaging properties of four different array detectors that are able to record THz radiation directly. Two micro-bolometer arrays are designed for infrared imaging in the 8–14 μm wavelength range, but are based on different absorber materials (i) vanadium oxide; (ii) amorphous silicon; (iii) a micro-bolometer array optimized for recording THz radiation based on silicon nitride; and (iv) a pyroelectric array detector for THz beam profile measurements. THz wavelengths of 96.5 μm, 118.8 μm, and 393.6 μm from a powerful far infrared laser were used to assess the technical performance in terms of signal to noise ratio, detector response and detectivity. The usefulness of the detectors for beam profiling and digital holography is assessed. Finally, the potential and limitation for real-time digital holography are discussed.

## 1. Introduction

Imaging with scanning methods is based on scan mirrors or translation stages for displacing the beam or the object, respectively. In contrast, imaging with array detectors allows for dynamic measurements, provided the detector is sensitive enough to terahertz (THz) radiation. In recent works on THz imaging and THz holography, micro-bolometer arrays were used as THz detectors. Bolometers are thermal detectors like pyroelectric detectors, Golay cells [1] and bi-material micro-cantilevers [2]. Indirect thermal methods record the temperature increase of a plate induced by absorption of THz radiation. This is based on thermography cameras or on thermally sensitive phosphor plates, which are read out with a CCD-camera [3]. For many practical applications, the scene imaged by the thermal detector is at room temperature. Therefore, the noise content of the image is dominated by the thermal radiation noise. As this thermal noise from the scene cannot be reduced substantially by using a cooled detector, uncooled array detectors are attractive. Uncooled micro-bolometer arrays for thermal imaging started to be used in the THz range around 2007 [4,5,6] after a first thorough study at the U.S. Naval Research Lab (NRL) [7]. A review on the use of uncooled bolometer-type infrared detectors for real-time THz imaging has been given by Oda [8] and Dem’yanenko [9]. Moreno *et al.* have compared various micro-bolometer absorber material systems [10]. While the main application of uncooled micro-bolometer arrays is thermal imaging, some arrays are optimized for detecting THz radiation. The latest developments of micro-bolometer arrays for use in the THz range above 0.6 THz, *viz.* wavelengths shorter than 500 μm, include cameras from the National Optics Institute (INO, Québec, Canada) [11], the NEC Corporation (Tokyo, Japan) [12], and the French Alternative Energies and Atomic Energy Commission (CEA Leti) [13,14]. Very recently a non-thermal large-size two-dimensional detector based on a commercial Si CCD has been reported for the visualization of intense THz pulses [15,16]. The CCD sensor shows a great potential for imaging applications demanding high spatial resolution. The visualization process, however, is nonlinear.

In this contribution, we focus on the applicability of thermal detector arrays to digital THz holography in an off-axis configuration, especially on real-time applications. We compare four detector arrays in a common experimental set-up, analyze their signal-to-noise ratio for applications of beam profiling and off-axis holography, and discuss the limits for real-time holography.

## 2. Figures of Merit

One basic figure of merit is the responsivity of the detector element to the power of the incoming radiation. Voltage responsivity [8] *R_V_* is defined as the ratio of the pixel output signal *V_S_* and the incident radiant power *P_0_* and is given in (V/W):

(1) $$R_{V}=\frac{\;V_{S}}{P_{0}}$$

For a micro-bolometer element built of an absorbing membrane coupled to a heat sink, the responsivity depends on the total absorptance of the element, the temperature coefficient of resistance (TCR) of the membrane material, and the thermal conductance of the coupling to the heat sink. Responsivity alone is, however, not sufficient for a comparison of detectors with different working principles. More meaningful parameters include the noise contributions, such as Signal-to-Noise Ratio (SNR), Noise Equivalent Temperature Difference (NETD), or Noise Equivalent Power (NEP). NEP is defined as the incident power that gives a signal-to-noise ratio of one. From Equation (1), NEP is given by

(2) $$NEP=\frac{\;V_{noise}}{R_{V}}$$

If a one Hertz output bandwidth is assumed, which is equivalent to half a second of integration time, NEP is given in $W/\sqrt{\text{Hz}}$. Typical NEP values for uncooled micro-bolometer arrays for thermal imaging are in the range of 200–300 $\text{pW}/\sqrt{\text{Hz}}$ [8,17].

The detectivity D* is often reported, which incorporates the measurement bandwidth and the area of the detector element. The detectivity relates to the NEP and is given in $\text{cm}\sqrt{\text{Hz}}/W$ by [1]

(3) $$D^{*}=\frac{\sqrt{A_{pix}B}}{NEP}$$

where *A_pix_* is the area of the detector element and *B* is the measurement bandwidth which is sometimes included in the NEP value as explained above.

For an uncooled thermal detector, it is common to state the NETD as the figure of merit which is defined as the temperature increase of a blackbody that increases the signal to noise ratio of the detector by one [1]. The NETD of a thermal camera includes the 1/f and Johnson noise of the bolometer, the thermal fluctuation noise, as well as the read-out noise [18]. It further depends on the optics and the wavelength range of operation. Since in digital holography cameras are used without optics, the reduced expression for the NETD is appropriate:

(4) $$NETD\;=\;\frac{NEP}{A_{pix}\times {\left(dE_{e,\lambda }/dT\right)\;|}_{\left[\lambda_{1},\lambda_{2}\right]}}$$

For the long wave infrared range (LWIR) micro-bolometers used in our work, the dependence of the spectral irradiance *E_e,λ_* on the temperature is evaluated in the wavelength band [8 μm, 14 μm] which yields ${\left(dE_{e,\lambda }/dT\right)\;|}_{\left[8,14\right]}=2.62\;{\text{Wm}}^{-2}K^{-1}$ at room temperature [1]. Equations (3) and (4) are used to calculate D* as appropriate, see Table 1.

**Table 1:** Specifications according to data sheets of the terahertz (THz) detectors compared in this work. D* is calculated using Equations (3) and (4) as appropriate. Range figures in brackets are calculated.

Detector Type	LWIR-Bolometer	LWIR-Bolometer	THz Bolometer	Pyroelectric Camera
Product	Devitech IR-032	XENICS Gobi 640	NEC IRV-T0831	Spiricon Pyrocam III HR
Identification	Cam1	Cam2	Cam3	Cam4
Detector material	VOx	a-Si	SiN	LiTaO_3_
Designated operation range	8–14 μm (22–37 THz)	8–14 μm (22–37 THz)	(43–300 μm) 1–7 THz	1.06–3000 μm 0.1–300 THz
Pixel size (μm)	NA	NA	NA	75
Pixel pitch (μm)	25	17	23.5	80
Number of pixels	640 × 480	640 × 480	320 × 240	160 × 160
Detector size (mm^2^)	16.0 × 12.0	10.9 × 8.2	7.5 × 5.6	12.8 × 12.8
NEP ($\text{nW}/\sqrt{\text{Hz}}$)	NA	NA	<0.1 @ 4 THz	12.8
NETD (mK)	50	50	NA	NA
Sensitivity	NA	NA	NA	96 nW/pix
D* ($\text{cm}\sqrt{\text{Hz}}/W$)	3.05 × 10^7^	4.49 × 10^7^	2.35 × 10^7^	5.86 × 10^7^
ADC (bit)	14	16	14	16
Frame rate (fps)	50	50	30	50

While the noise level can be estimated from a set of nominally equal images, the signal level needs to be defined for the practical situation at hand in order to calculate a meaningful signal-to-noise ratio. For a beam profiling instrument, the signal is obtained from the position of the maximum beam intensity; for interferometric measurements, the signal corresponds to the contrast of the interference pattern. In turn, for real-time imaging, a minimum useful SNR may be defined from which one may calculate either the limiting integration time or frame rate for a given THz power, or *vice versa*. Other factors influencing the performance of a detector array in digital off-axis holography are the number and size of pixels, which limit the lateral resolution in object space.

To take into account drifts of the laser power during the experiment as well as different integration times of the cameras, the SNR that is measured, $SNR_{meas}$, is transformed into a $SNR_{ref}$ for a reference power level *P_ref_* and a reference integration time *τ_ref_* according to

(5) $$SNR_{ref}\;=SNR_{meas}\frac{P_{ref}}{P_{meas}}\sqrt{\frac{\tau_{ref}}{\tau_{meas}}}$$

Equation (5) reflects the fact that the signal level is proportional to the THz power, while the noise level is reduced with the square root of the integration time or the square root of the number of averaged images. Note that the latter case is applied to the pyroelectric detector, as this instrument works in chopped mode.

## 3. Experimental

### 3.1. Thermal Array Detectors

Two thermal LWIR micro-bolometers with different absorbing layer materials, VOx (Devitech IR-032) and a-Si (XENICS Gobi 640), are compared to a THz micro-bolometer (NEC IRV-T0831) and a pyroelectric array camera (Pyrocam III HR with a THz transparent window made of low density polyethylene (LDPE)). The specifications of the cameras are summarized in Table 1.

### 3.2. THz Laser Source

A far infrared gas laser system FIRL-100 (Edinburgh Instruments Ltd, Edinburgh, UK) was used as the THz source, which includes a CO_2_ pump laser with a maximum of 60 W of single line output power. Methanol CH_3_OH or formic acid HCOOH was evaporated into the far infrared laser cavity as the lasing medium. Three THz wavelengths were selected to assess the detector performances across the THz range by tuning CO_2_ emission lines to pump the respective gas emission lines. The methanol line at 118.8 μm (rated maximum power 150 mW) and the formic acid line at 393.6 μm (40 mW), corresponding to 2.53 and 0.76 THz, respectively, were used to document the variation of the responsivity at two distinct points of the THz spectrum, while the methanol line at 96.5 μm (3.11 THz, 90 mW) was used to assess changes in performance around 3 THz. The diameter of the beam at the laser exit port is 10 mm (1/e^2^ point).

### 3.3. Experimental Set-Up

**Figure 1:**
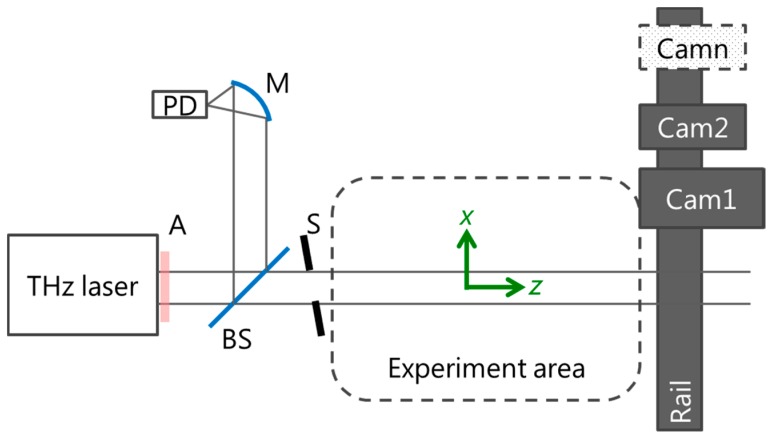
Experimental set-up, schematic. A: Teflon absorber plate; PD: Pyroelectric detector; M: Focusing Mirror; BS:; 10/90 beam splitter; S: shutter; Camn: Camera #n.

In order to realize reproducible measurement conditions, all four cameras were mounted onto a common rail and aligned in such a way that the array detectors were located on a single plane perpendicular to the laser beam, see Figure 1. The THz power was controlled with an independent pyroelectric detector mounted in the low power arm of a beam splitter plate. As only the comparison of detectors was of interest in this work, the absolute THz power reaching the detector was not measured. A shutter was inserted into the beam in order to allow measurement and subtraction of the infrared background radiation. In order to prevent a saturation of the camera, the THz beam was attenuated by inserting Teflon plates of appropriate thickness at the laser exit port.

**Figure 2:**
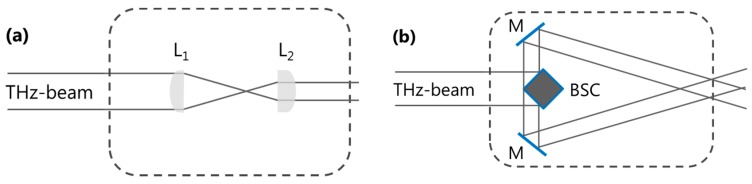
Experiment area, schematic. (**a**) beam collimation with lenses L_1_ and L_2_; (**b**) two-beam interference, based on a surface reflecting beam splitter cube (BSC) and two mirrors (M).

Figure 2 shows the two set-ups used in the experiment area. For the measurement of the beam profile and the detector homogeneity, the THz laser beam was collimated with a pair of Tsurupica lenses with 100 mm and 50 mm focal length, respectively, to a diameter of 5 mm at the detector plane, Figure 2a. In the set-up of Figure 2b, the THz laser beam was split by using a polished steel cube and recombined through a set of two mirrors such that the beam overlap was located at the detector plane. This arrangement was used for the alignment of the cameras and for providing a regular interference pattern. The alignment of the cameras was achieved by sequentially illuminating a pin placed in the beam overlap area in front of the camera. The camera was then displaced along the beam propagation *z* direction such that the distance between the two diffraction patterns on the detector plane was the same for all cameras.

#### 3.3.1. Camera Response

The detector plane of the camera was centered on the collimated THz laser beam, Figure 2a. Care was taken not to saturate the detector by inserting Teflon blocks of appropriate thickness. For a better comparability, images were scaled to the effective THz power measured. While this result is sufficient to compare the performance of the cameras when used as a beam profiler, for THz holography, the full area of the detector is used, and variations of the camera response across the image are important. It has to be noted that with coherent radiation the image does not only depend on detector responsivity, Equation (1), but also on other camera components, e.g., the cover plate or the housing. To assess this variation, the cameras were displaced in regular intervals in two directions through the beam and an image was recorded at each position. A combination of the images allowed extracting a relative camera response map as well as a corrected THz image.

#### 3.3.2. Interference Fringe Contrast

The two-beam interferometer set-up, Figure 2b, was used to generate an interference pattern with a regular fringe spacing of Λ = 0.7 mm in horizontal *x*-direction for the 118.8 μm wavelength in the overlap region. The fringe spacing was adjusted such that all cameras had a sufficient number of pixels across one fringe to reliably determine the modulation signal. The detector was placed into the overlapping beam area to record the interference fringe pattern which is written as

(6) $$I=I_{B}+I_{0}+I_{M}\cos\;\left(\frac{2\pi }{\Lambda }x+\phi_{0}\right)$$

where *I_B_* is the thermal background, *I_0_* the average THz intensity, *I_M_* the modulation intensity of the THz fringe pattern, and $\phi_{0}$ is a phase shift. An ideal sinusoidal pattern was extracted from the recorded fringe pattern. The signal level was identified with 2*I_M_*. The noise level was again obtained from the pixel fluctuations of consecutive images. Illuminating the entire detector reveals interference effects that can severely affect a hologram such as diffraction from the detector housing due to the coherent nature of the THz radiation.

#### 3.3.3. Real-Time Holography

The SNR obtained from the modulation intensity and the noise level of the interference pattern, forms the basis for a calculation of the capability of the cameras for real-time off-axis holography. If we define a real-time experiment to be a video-rate experiment, the frame rate is typically 25 fps. From the maximum SNR of the interference fringe pattern and the corresponding effective camera integration time, we obtain the SNR of a 40 ms integration time corresponding to an output bandwidth of 12.5 Hz. If, in addition, we set the limit for useful experiments at a value of SNR > 4, we can determine the minimum THz power at which this level is obtained, assuming linear integration on the camera, according to Equation (5).

While for real-time imaging the discussion of integration time *vs.* SNR is sufficient, for real-time holography, additional issues arise. Real time holography is based on the evaluation of amplitude and phase from a single hologram. In off-axis holography, this can be achieved using, e.g., a Fourier transform phase retrieval method [19]. Three conditions must be fulfilled: (i) the SNR of a single frame must be high enough to be evaluated; (ii) the limiting resolution of the digital holography set-up must be appropriate [20]; (iii) the Fourier peak of the carrier fringes must be well separated from the background peak.

The calculation of the limiting resolution involves the convolution with the resolution functions given by the pixel size *p* and the numerical aperture [21]. While the resolution function due to pixel size is a rectangle of width *p*, the intrinsic resolution function in Fresnel approximation takes the form of a sinc function with a first zero found at

(7) $$d_{W}=\lambda \frac{g}{N\;p}$$

where *λ* is the wavelength, *g* the reconstruction distance and *N* the number of pixels in the considered direction. Resolution increases with smaller pixel pitch *p* and larger detector size *Np*.

To evaluate the phase of the diffracted object wave using the Fourier transform phase retrieval method, it is necessary to separate the spectrum of the object wave around the carrier frequency in the Fourier plane from the image background found around the zero frequency peak. The carrier frequency is ideally located at half the maximum frequency, which guarantees a good separation from the zero frequency peak, but at the same time avoids aliasing effects. This frequency corresponds to a fringe spacing of Λ_min_ = 4*p* or a maximum angle α_max_ between the object and reference beam in the off-axis holography set-up given by

(8) $$\sin\;\alpha_{\max}=\frac{\lambda }{4\;p}$$

## 4. Results and Discussion

### 4.1. Beam Profiling

**Figure 3:**
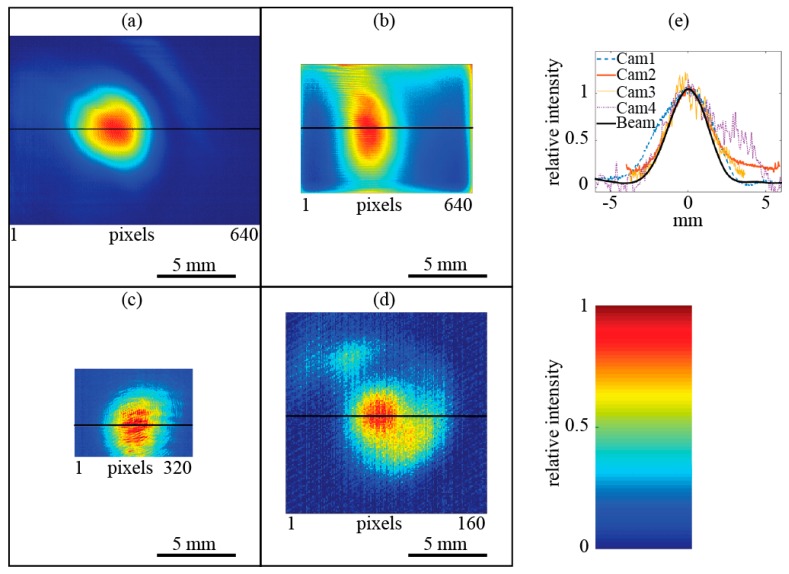
Beam profile at a wavelength of λ = 118.8 μm measured with the four cameras. (**a**) Cam1; (**b**) Cam2; (**c**) Cam3; (**d**) Cam4; (**e**) horizontal cross-section through the measured laser spots and the beam profile (black line).

Figure 3 shows a beam profile measured with the four cameras at λ = 118.8 μm. The image areas are scaled identically to represent the relative detector areas, such that the measured beam should have the same width. Note that the apparent shape of the beam is asymmetric, especially for Cam1 and Cam4, Figure 3e. This may be caused by the inhomogeneous response across the detector area of some cameras and will be discussed in Section 4.2 below. The SNR is calculated by dividing the average signal value at the beam center by the noise level. The noise level is determined from the variation of two consecutive images by averaging the squared difference of neighboring pixel values. For the micro-bolometer detectors (Cam1, Cam2 and Cam3), the noise is dominated by the fluctuations of the thermal background, while the THz radiation does not contribute appreciably. Values for the SNR are compiled in Table 2 for two wavelengths of λ = 118.8 μm and λ = 393.6 μm.

**Table 2:** Results of *SNR_ref_* for the cameras at a terahertz (THz) power of *P_ref_* = 10 mW and an integration time of *τ_ref_* = 25 ms. Numbers in brackets correspond to values obtained with an IR-filter in front of the camera.

Detector Type	LWIR-Bolometer	LWIR-Bolometer	THz Bolometer	Pyroelectric Camera
Identification	Cam1	Cam2	Cam3	Cam4
*SNR_ref_* for beam profile at λ = 118.8 μm	60	80	60	3
*SNR_ref_* for beam profile at λ = 393.6 μm	10	7	15	3
*SNR_ref_* for interference fringes at λ = 118.8 μm	85 (70)	50	60 (40)	0.5

### 4.2. Camera Response

**Figure 4:**
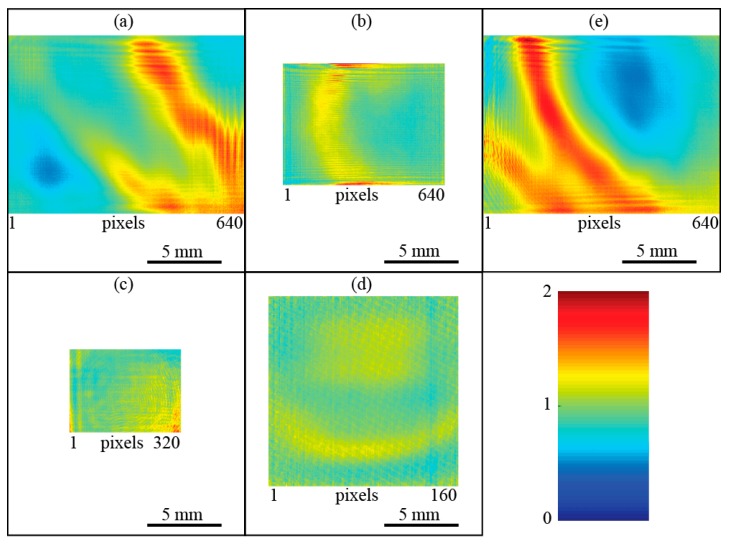
Relative camera response across the entire image area at λ = 118.8 μm (**a**–**d**). A value of one corresponds to the average response of the image area. (**a**) Cam1; (**b**) Cam2; (**c**) Cam3; (**d**) Cam4; (**e**) Cam1 at λ = 96.5 μm.

Figure 4a-d shows the camera response at λ = 118.8 μm relative to the average response as determined from the images of the beam at different positions on the detector. The diffraction effects from the borders are clearly visible and assessed in more detail in Section 4.3. There is a huge variation of the response across the detector area of Cam1, Figure 4a. As such a large variation cannot be the result of inhomogeneous detector responsivity, this effect is attributed to interference effects inside the camera, e.g., as a result from a varying distance between the detector array and the cover plate. To corroborate this hypothesis, the measurement of the camera response was repeated at a second close-by wavelength clearly showing a shift in the maxima and minima, Figure 4e. Cameras Cam2, Cam3 and Cam4 have a more homogeneous response.

### 4.3. Interference Fringe Pattern

**Figure 5:**
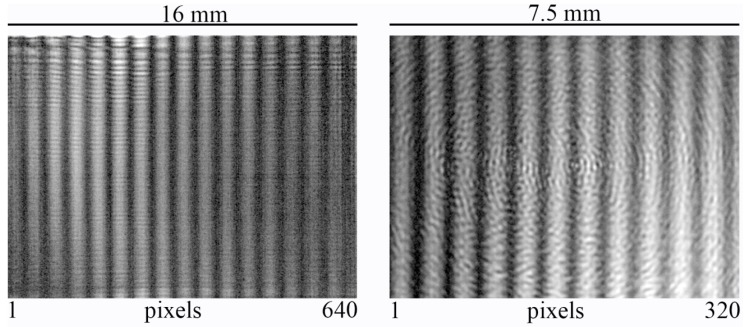
High frequency diffraction patterns caused by the camera housing interfering with the two-beam fringe pattern, measured at λ = 118.8 μm. Cam2 (**Left**) and Cam3 (**Right**).

The diffraction originating from scattering off the camera housing represents a systematic unwanted contribution to a hologram. Figure 5 represents typical patterns from two cameras featuring diffraction patterns from a linear and a circular edge, respectively. The image contrast has been scaled to highlight the diffraction patterns.

**Figure 6:**
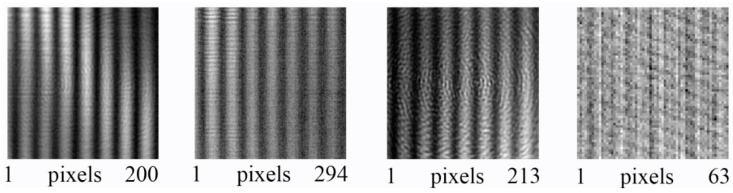
Central part (5 × 5 mm^2^) of the interference pattern recorded on Cam1 to Cam4 (**Left** to **Right**) at λ = 118.8 μm.

Figure 6 shows the central area with a size of 5 × 5 mm^2^ from the original fringe patterns recorded with the four cameras. In order to determine the SNR of these fringe patterns, we remove the high frequency noise caused by edge diffraction as well as the low frequency background to obtain ideal sinusoidal fringe patterns. From these, the local modulation intensity *I_M_* is determined according to Equation (6) and is divided by the noise level. The SNR values obtained for the reference values *P_ref_* = 10 mW and *τ_ref_* = 25 ms according to Equation (5) are compiled in Table 2 for a wavelength of λ = 118.8 μm. Note that a direct comparison of *SNR_ref_* for beam profile and interference fringes is not possible due to different experimental conditions. A considerable amount of THz power was lost in the lens system used for beam profiling, Figure 2a.

### 4.4. Real Time Off-Axis Holography

From the SNR of the interference fringe pattern and the corresponding effective camera integration time, we obtain the SNR of an equivalent 25 fps camera, *i.e.*, the SNR for a 40 ms integration time corresponding to an output bandwidth of 12.5 Hz, according to Equation (5). In turn, fixing the THz power to 10 mW and assuming a limiting SNR of 4 we obtain the necessary integration time; while fixing the integration time to 40 ms and SNR to 4 allows for calculating the necessary THz power. These values are shown in Table 3 together with the limiting interference angle enclosed by the reference and object wave according to Equation (8). As for all bolometer cameras, the relation 4p≤λ holds for λ = 118.8 μm, the maximum interference angle is unrestricted, while for Cam4 it is 22°. Note, however, that the housing may prevent using large angles due to excessive diffraction or obstruction from the housing.

**Table 3:** Use of the cameras in real-time holography and limitation on the interference angle, Equation (8).

Identification	Cam1	Cam2	Cam3	Cam4
SNR for 40 ms integration time and 10 mW THz power	136	80	96	0.8
Integration time for SNR = 4 and 10 mW THz power	0.035 ms	0.100 ms	0.069 ms	1000 ms
THz power for SNR = 4 and 40 ms integration time	0.29 mW	0.50 mW	0.42 mW	50 mW
$\alpha_{\max}$ λ = 118.8 μm	90°	90°	90°	22°
$\alpha_{\max}$ λ = 96.5 μm	75°	90°	90°	18°

**Figure 7:**
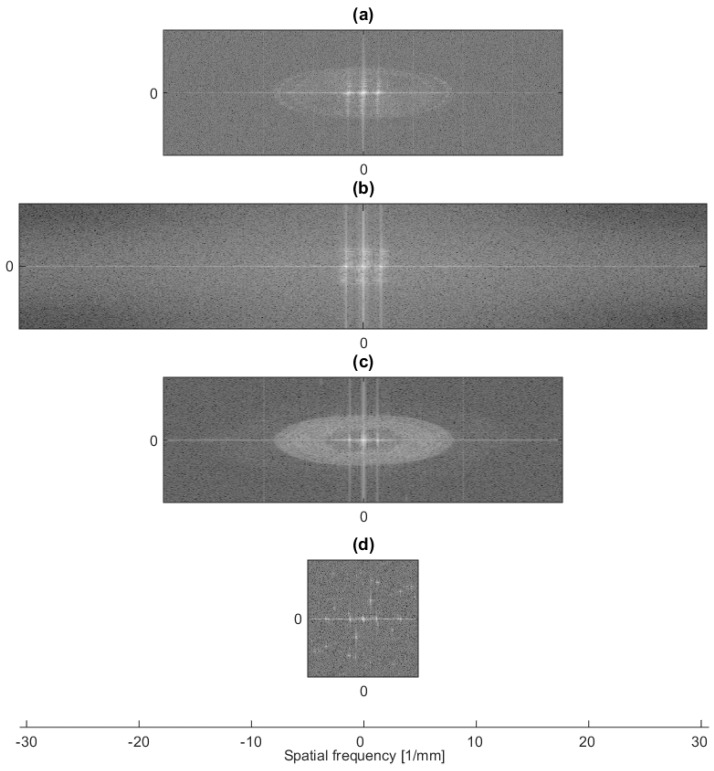
Fourier transform of the interference pattern recorded at λ = 118.8 μm. (**a**) Cam1; (**b**) Cam2; (**c**) Cam3; (**d**) Cam4.

Figure 7 shows the Fourier power spectrum of the interference pattern taken at λ = 118.8 μm for all cameras. Note that the *y*-axis has been compressed for convenience of display, while the spectral intensity is scaled to the intensity of the satellite peaks. While the zero peak and the two main satellites representing the sinusoidal fringe pattern at 1.4 mm^−1^ are clearly visible for Cam1 to Cam3, they are difficult to identify for Cam4 in Figure 7d. From Figure 7, it can be concluded that Cam1, Cam2 and Cam3 can accommodate much higher spatial frequencies than Cam4. Circular diffraction patterns exemplified in Figure 5 (right) give rise to the elliptic halos in Figure 7a,c.

**Figure 8:**
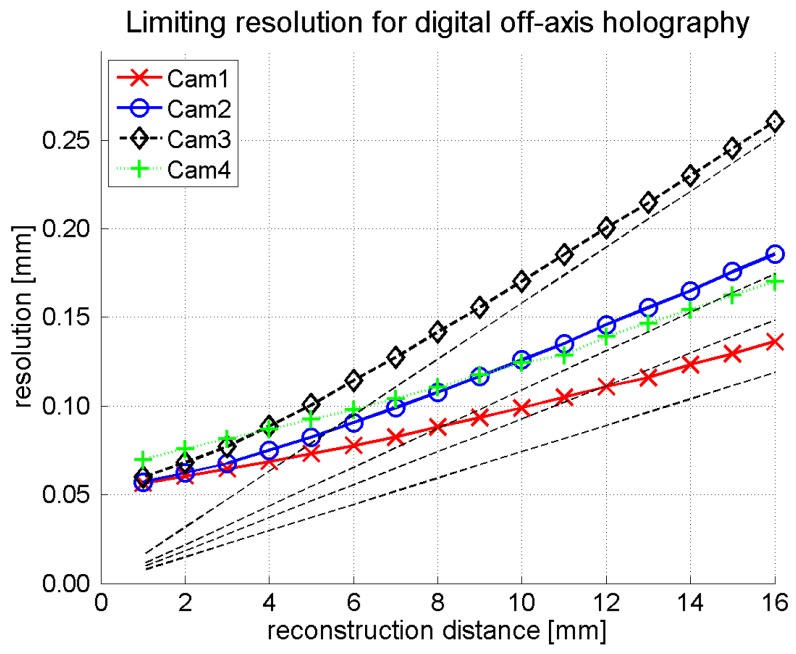
Radius of the reconstructed point source with a wavelength of 118.8 μm for a set of source distances. The dotted lines represent the resolution in Fresnel approximation as described by Equation (7).

An estimate of the limiting resolution expected in digital holography as a function of the reconstruction distance is represented in Figure 8 for all cameras for the long detector axis (*x*-axis). In this simulation, a point source at a distance from the detector plane and an off-axis reference wave with a wavelength of 118.8 μm and an angle of 22° were assumed. The interference pattern was resampled on the pixel grid of the respective camera. The point source was then reconstructed using the Kirchhoff theory without approximation yielding sinc-like functions. The curves in Figure 8 represent the distance of the first minimum from the central peak. The dotted lines represent the resolution in Fresnel approximation as described by Equation (7).

## 5. Conclusions/Outlook

All four cameras assessed in this work can be used for THz imaging and real-time THz holography, if sufficient THz power is available. More care must be taken when using thermal micro-bolometers. It has been shown that the camera response may not be homogeneous across the detector area because of uncontrollable interference effects between the detector plane and the cover plate. In turn, the dedicated THz micro-bolometer and pyroelectric detector do not show an appreciable variation of the camera response across the detector area. The SNRs for all three bolometers are comparable, whereas the pyroelectric camera has significantly lower SNRs around 3 THz. This is also reflected in the calculated D* values that are within 2.3 and 4.5 × 10^7^ $\text{cm}\sqrt{\text{Hz}}/W$ for the bolometers, while the pyroelectric camera is a factor of 40 less sensitive. Note that the D* value of the LWIR-bolometers was calculated from the specifications given in the 8 to 14 μm infrared band.

Dedicated THz cameras do not yet reach the high lateral resolution of the thermal micro-bolometers both in number of pixels and in pixel pitch. Currently, they are not well suited for real-time THz holography. On the other hand, an LWIR bolometer may suffer from inhomogeneous response due to interference effects. Special care has to be taken of interference effects due to the camera housing. Additional measurements (with and without samples) or synthetic aperture methods with overlapping camera positions can be used to suppress such artefacts.
